# Melatonin Inhibits Reactive Oxygen Species-Driven Proliferation, Epithelial-Mesenchymal Transition, and Vasculogenic Mimicry in Oral Cancer

**DOI:** 10.1155/2018/3510970

**Published:** 2018-03-21

**Authors:** Rui Liu, Hui-li Wang, Man-jing Deng, Xiu-jie Wen, Yuan-yuan Mo, Fa-ming Chen, Chun-li Zou, Wei-feng Duan, Lei Li, Xin Nie

**Affiliations:** ^1^Department of Stomatology, Daping Hospital and Research Institute of Surgery, Third Military Medical University, Chongqing 400042, China; ^2^School and Hospital of Stomatology, Wenzhou Medical University, Wenzhou, Zhejiang 325035, China; ^3^Department of Infection and Immunity, State Key Laboratory of Trauma, Burn and Combined Injury, Daping Hospital and Research Institute of Surgery, Third Military Medical University, Chongqing 400042, China; ^4^Department of Nursing, Xi'an International University, Xi'an 710077, China; ^5^State Key Laboratory of Military Stomatology and National Clinical Research Center for Oral Diseases, Department of Periodontology, School of Stomatology, Fourth Military Medical University, Xi'an 710032, China; ^6^Department of Maxillofacial Surgery, Daping Hospital and Research Institute of Surgery, Third Military Medical University, Chongqing 400042, China; ^7^Department of Stomatology, 8th Hospital of People's Liberation Army, Shigatse 857000, China

## Abstract

Globally, oral cancer is the most common type of head and neck cancers. Melatonin elicits inhibitory effects on oral cancer; however, the biological function of melatonin and underlying mechanisms remain largely unknown. In this study, we found that melatonin impaired the proliferation and apoptosis resistance of oral cancer cells by inactivating ROS-dependent Akt signaling, involving in downregulation of cyclin D1, PCNA, and Bcl-2 and upregulation of Bax. Melatonin inhibited the migration and invasion of oral cancer cells by repressing ROS-activated Akt signaling, implicating with the reduction of Snail and Vimentin and the enhancement of E-cadherin. Moreover, melatonin hampered vasculogenic mimicry of oral cancer cells through blockage of ROS-activated extracellular-regulated protein kinases (ERKs) and Akt pathways involving the hypoxia-inducible factor 1*α*. Consistently, melatonin retarded tumorigenesis of oral cancer *in vivo*. Overall, these findings indicated that melatonin exerts antisurvival, antimotility, and antiangiogenesis effects on oral cancer partly by suppressing ROS-reliant Akt or ERK signaling.

## 1. Introduction

Oral cancer is one of the most common malignancies worldwide [1]. Oral squamous cell carcinoma is the most prevalent type and accounts for 90% of oral cancers [2]. Despite great advances in surgical treatment, radiotherapy, and chemotherapy, the high reoccurrence and poor prognosis of oral cancer appear frequently owing to the rapid growth, local invasiveness, and distant metastasis [3–5]. Thus, exploring the underlying mechanisms of oral cancer growth and metastasis and identifying a potential therapeutic agent for oral cancer are imperative.

Reactive oxygen species (ROS) are highly associated with several pathological conditions, including cancers [6]. It is established that cancer cells usually generate a large amount of ROS [7]. ROS can activate Akt signaling responsible for the proliferation and apoptosis evasion in several cancers, including oral cancer [8, 9]. ROS are highly involved in cancer metastasis, which are proposed to be putative mediators or modulators of epithelial-mesenchymal transition (EMT) [10–12]. EMT plays important roles in cancer metastasis and mainly endows cancer cells with invasive phenotypes [13]. During EMT, epithelial cells lose their epithelial markers, such as E-cadherin, and gain the mesenchymal markers, including Vimentin and Snail [14]. Reportedly, Snail is a transcriptional repressor of E-cadherin in tumor cells [15]. Mechanistically, the activation of phosphatidylinositide 3-kinases (PI3K)/Akt pathway is essential for ROS-driven EMT in colon cancer cells; in detail, ROS-dependent Akt activation enhances the expression of Snail and Vimentin and simultaneously reduces E-cadherin expression [16]. ROS is also a critical regulator of angiogenesis that is essential for cancer metastasis [17, 18]. ROS induces the expression of hypoxia-inducible factor 1*α* (HIF-1*α*) and increases its stabilization, thereby leading to the upregulation of vascular endothelial growth factor (VEGF) and subsequent angiogenesis [19, 20]. Both extracellular-regulated protein kinases (ERK) and PI3K/Akt pathways greatly contribute to the ROS-enhanced HIF-1*α* and VEGF during the malignant transformation of human bronchial epithelial cells [21]. Thus, targeting ROS is a potential therapeutic strategy to reduce the malignant phenotypes of oral cancer.

Melatonin (N-acetyl-5-methoxytryptamine), which is mainly produced and secreted by the pineal gland, was initially found to be involved in the regulation of chronobiological rhythm [22]. Recently, as a potent antioxidant, melatonin scavenges a variety of free radicals directly and stimulates the activities of antioxidative enzymes indirectly [23, 24]. Melatonin exerts suppressive effects on multiple types of tumors partly depending on free radical scavenging and antioxidative activities [25–27]. Previously, melatonin was utilized as a preventive and curative agent for oral cancers by abolishing oxidative stress [28, 29]. Nevertheless, whether melatonin hampers oral cancer by reducing ROS-increased proliferation, EMT, and angiogenesis remains unknown.

In this study, we first found that oral cancer cells highly produced ROS, which were significantly decreased by melatonin treatment. Melatonin reduced the proliferation and apoptosis resistance in oral cancer cells by inactivating ROS-dependent Akt signaling. The antimigratory and anti-invasive effects of melatonin on oral cancer cells were dependent on the suppression of EMT via inactivation of ROS-reliant Akt signaling. The antiangiogenic effect of melatonin on oral cancer cells was through the blockage of ROS-activated ERK and Akt pathways. Consistently, melatonin retarded the carcinogenesis of oral cancer *in vivo*. Overall, melatonin repressed the proliferation, motility, and angiogenesis of oral cancer cells partially by repressing ROS-activated ERK and Akt signaling.

## 2. Materials and Methods

### 2.1. Materials

Human umbilical vein endothelial cells (HUVECs) and six human oral cancer cell lines, including SCC25, SCC9, Tca8113, Cal27, and FaDu, were obtained from American Type Culture Collection (Manassas, VA, USA). Human normal oral keratinocytes (hNOKs) were purchased from ScienCell Research Laboratories (Carlsbad, CA, USA). M200 medium was acquired from Cascade Biologics (Portland, OR, USA). Dulbecco's modified Eagle's medium (DMEM) and fetal bovine serum (FBS) were provided by Gibco (Carlsbad, CA, USA). Oral keratinocyte medium (OKM) was supplied by ScienCell Research Laboratories. 2′,7′-Dichlorofluorescein diacetate (DCFH-DA) was obtained from Molecular Probes (Eugene, OR, USA). Antibodies against phosphorylated (p)-Akt (Ser473), Akt, p-ERK1/2 (Thr202/Tyr204), and ERK1/2 were obtained from Cell Signaling Technology (Beverly, MA, USA). Antibodies targeting Snail, E-cadherin, and Vimentin were acquired from Santa Cruz Biotechnology (Santa Cruz, CA, USA). Antibodies against cyclin D1, proliferating cell nuclear antigen (PCNA), Bax, Bcl-2, HIF-1*α*, VEGF, and glyceraldehyde-3-phosphate dehydrogenase (GAPDH) were from Abcam (Cambridge, UK). Horseradish peroxidase (HRP)-conjugated and Cy3-conjugated secondary antibodies were acquired from BD Biosciences (San Diego, CA, USA). Akt inhibitor LY294002, ERK1/2 inhibitor U0126, ROS scavenger N-acetyl-L-cysteine (NAC), and melatonin were purchased from Sigma-Aldrich (St. Louis, MO, USA). All other reagents were from Sigma unless otherwise stated.

### 2.2. Cell Culture and Treatment

HUVECs were cultured in M200 medium with low-serum supplements (Cascade Biologics), penicillin (100 U/mL), and streptomycin (100 *μ*g/mL). Oral cancer cells were cultured in DMEM supplemented with 10% FBS, 100 U/mL penicillin, and 100 *μ*g/mL streptomycin. hNOKs were cultivated in OKM. All the cells were cultured in a humidified incubator with 5% CO_2_ at 37°C. For melatonin treatment, confluent Cal27 and FaDu cells were exposed to melatonin (1 mM) dissolved in dimethyl sulfoxide (DMSO; 0.2% final concentration) for 24 h. To investigate the suppressive effects of NAC, LY294002, and U0126, Cal27 and FaDu cells were treated with or without NAC (5 mM), LY294002 (20 *μ*M), or U0126 (20 *μ*M) until they were harvested for analyses.

### 2.3. Measurement of ROS Production

ROS generation was measured by using DCFH-DA assay. Briefly, Cal27 and FaDu cells were seeded in six-well plates and treated as described in Section 2.2. Tumor tissues were homogenized and centrifuged, and the supernatants were collected. Afterwards, 10 *μ*M of DCFH-DA was added to the cells or supernatants and incubated for 30 min at 37°C. DCF fluorescence was determined at 488 nm excitation and 525 nm emission using a fluorescence microplate reader (Bio-Rad, Hercules, CA, USA).

### 2.4. Cell Viability Assay

The 3-(4,5-dimethylthiazol-2-yl)-2, 5-diphenyl-tetrazolium bromide (MTT) assay was performed to measure cell viability. In brief, Cal27 and FaDu cells were seeded in a 96-well plate at a concentration of 1 × 10^3^ cells/well and cultured for 24 h. After different treatments, the cells were cultured for 1, 2, 3, or 4 days. MTT (0.5 mg/mL) was added in each well and incubated at 37°C for 4 h. The supernatants were then removed, and DMSO (0.15 mL) was added to each well. The absorbance was measured at 490 nm by using a microplate reader (Bio-Rad).

### 2.5. Colony Formation Assay

Cell proliferation was assessed by colony formation assay. Briefly, Cal27 and FaDu cells (1 × 10^3^) were seeded into six-well plates and maintained in culture medium containing 10% FBS, which was refreshed every two days. After being incubated for 14 days at 37°C in 5% CO_2_, the colonies were fixed with methanol and stained with 1% crystal violet for 15 min and their numbers were counted using ImageJ software (NIH, USA).

### 2.6. Determination of Apoptosis by Flow Cytometry

Cell apoptosis was determined using Annexin V/Fluorescein isothiocyanate (FITC) and propidium iodide (PI) Apoptosis Detection Kit (BD Biosciences, San Diego, CA, USA) following the manufacturer's protocol. In brief, after different treatments, Cal27 and FaDu cells were harvested, centrifuged, and resuspended in 100 mL of 1 × binding buffer at a final concentration of 1 × 10^6^ cells/mL. Then, 10 *μ*L of ready-to-use Annexin V/FITC was added in the mixture and incubated at 37°C for 15 min followed by 5 *μ*L of PI counterstaining in the dark for 30 min. The Annexin V/FITC and PI fluorescence were assessed by BD FACSCalibur flow cytometry (BD Biosciences). Results were analyzed by CellQuest software (BD Biosciences).

### 2.7. Nucleosomal Fragmentation Assay

Cell apoptosis was detected using a Cell Death Detection ELISA PLUS kit (Roche Diagnostics, Germany) according to the manufacturer's instructions. Briefly, after different treatments, Cal27 and FaDu cells (1 × 10^4^) were lyzed and the nuclear fractions served as an antigen source in a sandwich ELISA, utilizing primary antihistone antibody-coated microplate and a secondary peroxidase-conjugated anti-DNA antibody. The photometric immunoassay for histone-associated DNA fragments was executed at 405 nm using a microplate reader (Bio-Rad). The absorbance values were normalized to those from control-treated cells.

### 2.8. Migration and Invasion Assays

Transwell chambers (8-*μ*m pore size; Corning Costar, Dallas, TX, USA) with or without Matrigel were used to assess the migratory or invasive ability of Cal27 and FaDu cells, respectively. For migration assay, the cells with or without melatonin or NAC treatment were seeded into the upper chamber and cultured in serum-free DMEM. The lower chamber was filled with DMEM containing 15% FBS as a chemoattractant. For invasion assay, except for the bottom of the upper chamber precoated with Matrigel (200 *μ*g/mL; BD Biosciences, San Jose, CA), the protocol was the same with that in the migration assay. After incubation for 24 h at 37°C, the cells on the upper layer of the chambers were removed and the cells migrated or invaded through the membrane were fixed with methanol and stained with crystal violet. Five fields of each membrane were observed and photographed under a fluorescence microscope (Olympus, Tokyo, Japan). The average number of migrated or invaded cells in every field of each well was calculated.

### 2.9. Immunofluorescence Staining Assay

Cal27 and FaDu cells subjected to the indicated treatments were seeded into six-well plates with sterile glass coverslips. Cells on the coverslips were fixed with 4% paraformaldehyde for 20 min, permeabilized with 0.2% Triton X-100 for 10 min, washed with PBS twice, and blocked with 0.5% bull serum albumin for 30 min at room temperature. Then, the coverslips were incubated with primary antibodies against E-cadherin, Vimentin, and Snail overnight at 4°C, followed by incubation with Cy3-conjugated secondary antibodies for 30 min at 37°C in the dark. Nuclei were counterstained with 4′,6-diamidino-2-phenylindole (Sigma). Images were captured with a fluorescence microscope (Olympus).

### 2.10. Western Blot Analysis

Cal27 and FaDu cells and the tumor tissues were lysed in lysis buffer (Beyotime Biotechnology, Shanghai, China). The extracted proteins were separated by sodium dodecyl sulfate-polyacrylamide gel electrophoresis and transferred onto polyvinylidene-fluoride membranes (Bio-Rad). The membrane was blocked with 5% nonfat dry milk in Tris-buffered saline and Tween 20 (TBST) for 1 h at room temperature and incubated with primary antibodies against p-Akt, Akt, cyclin D1, PCNA, Bax, Bcl-2, Snail, E-cadherin, Vimentin, p-ERK, ERK, HIF-1*α*, VEGF, and GAPDH at 4°C overnight. After washing with TBST thrice, the membranes were incubated with appropriate HRP-conjugated secondary antibodies for 1 h at 37°C and visualized using enhanced chemiluminescence kit (Thermo Scientific, Rockford, IL, USA). The band density was quantified using the Quantity One software (Bio-Rad). The Western blot results were normalized with the internal control GAPDH and then calculated as fold changes compared with the first treatment group (fold of basal is 1.00) in each experiment.

### 2.11. Tube Formation Assay

Angiogenesis was assessed by tube formation assay. After 24-well plates were coated with 200 *μ*L of Matrigel at a concentration of 1 mg/mL, HUVECs (4 × 10^4^) were seeded into each well and cultured in M200 medium containing 50 *μ*L of freshly collected conditioned medium (CM) from Cal27 and FaDu cells with different treatments. Following a 36 h incubation period, the tubules were observed and photographed under a phase contrast microscope (Olympus). The number of the tubules was calculated.

### 2.12. Measurement of VEGF Production

VEGF generation was measured using enzyme-linked immunosorbent assay (ELISA) with a commercial kit (R&D Systems, Minneapolis, MN, USA). Cal27 and FaDu cells were seeded into six-well plates at a density of 1 × 10^5^ and treated as described in Section 2.2. Then, the culture media were collected for VEGF measurement. 200 *μ*L of cell supernatants or standards was added to the microplate precoated with monoclonal antibody against VEGF and incubated for 2 h at room temperature, followed by treatment with HRP-conjugated second antibody for 2 h at room temperature. Substrate solution (200 *μ*L) was put in each well and incubated for 30 min, and stop solution (50 *μ*L) was added to stop the reaction. The optical density was measured at 450 nm on a microplate reader (Bio-Rad).

### 2.13. Xenograft Tumor Model

Eight-week-old male SCID mice (weighing from 22 g to 25 g) were purchased from the Institute of Zoology, Chinese Academy of Sciences (Beijing, China) and maintained under specific pathogen-free conditions at 22°C to 24°C in relative humidity of 40%–60% and a regular 12 h day/night cycle with free access to food and water. All the experimental procedures were approved by the Institutional Guidelines of Animal Care and Use Committee of Third Military Medical University.

All mice received subcutaneous injection of 2 × 10^6^ Cal27 cells and then were randomly allocated into control and melatonin groups (*n* = 6 per group). Control group mice were intraperitoneally injected with 100 *μ*L of vehicle solution (PBS). Animals in the melatonin group received intraperitoneally 100 *μ*L of melatonin at 40 mg/kg of body weight. Melatonin was administered 1 h before room lighting was switched off. Melatonin or vehicle injection started on the 7th day of tumor cell implantation (tumor volume = 80 mm^3^) and was performed for five days a week in a span of three weeks. Tumor size was measured once a week using the caliper, and tumor volume was calculated using the formula: tumor volume = (length × width^2^)/2. Six weeks later, the mice were euthanized, and the tumors were removed and weighed. The tumor tissues were prepared for ROS measurement, Western blot analysis, and immunohistochemistry (IHC) assay.

### 2.14. IHC Assay

Tumor tissues were sectioned, mounted on to slides, deparaffinized with xylene, and dehydrated in a series of graded ethanol. For antigen retrieval, the sections were boiled in citric buffer (pH 6.0) for 10 min using a hot plate and then cooled for 1 h at room temperature. Endogenous peroxidase was blocked with 3% H_2_O_2_ for 15 min, and the sections were blocked with 10% normal goat serum for 20 min. Antibodies against p-Akt, p-ERK, E-cadherin, Vimentin, Snail, cyclin D1, PCNA, Bax, Bcl-2, HIF-1*α*, and VEGF were applied overnight at 4°C, followed by incubation with secondary antibodies for 1 h at 37°C. Sections were then stained with freshly prepared diaminobenzidine substrate (Maixin Biotech., Fuzhou, China), counterstained with hematoxylin, dehydrated, and mounted. Five nonoverlapping fields per slide were randomly selected, and images were captured with a light microscope attached to a digital camera (Olympus). The captured images were examined independently by two experienced pathologists in a blinded manner.

### 2.15. Statistical Analyses

All the data were expressed as mean ± SD. Student's *t*-test or one-way ANOVA analysis, followed by Dunnett's post hoc test was used to determine the differences between two groups or in multiple groups. Statistical analyses were performed using GraphPad Prism (GraphPad Software Inc., San Diego, CA, USA). *P* < 0.05 was considered as statistical significance.

## 3. Results

### 3.1. Melatonin Inhibits ROS Production in Oral Cancer Cells

**Figure 1:**
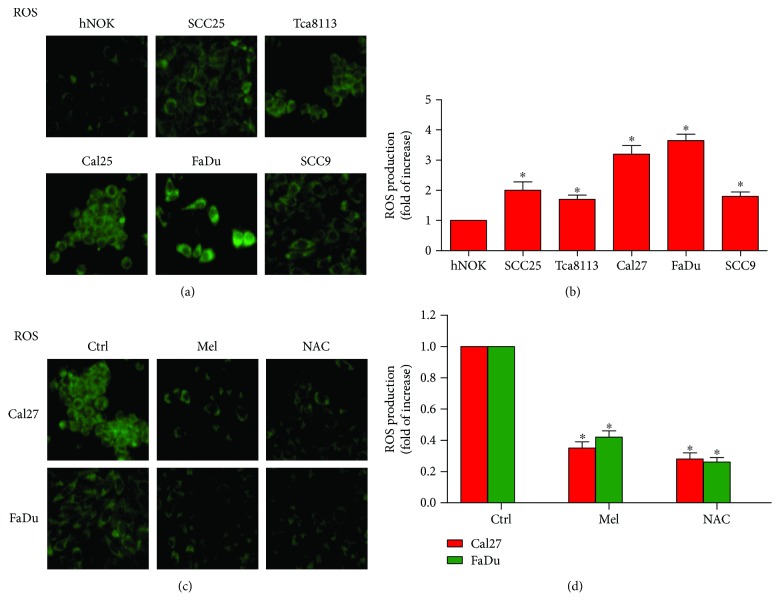
Melatonin reduced ROS production in oral cancer cells. (a) The levels of ROS in hNOKs and five oral cancer cell lines including SCC25, SCC9, Tca8113, Cal27, and FaDu were determined by DCFH-DA assays. Magnification: 200x. (b) Relative quantification of ROS in (a) was calculated. (c) Cal27 and FaDu cells were seeded in six-well plates and treated with melatonin (1 mM), NAC (5 mM), or the vehicle (control) for 24 h. DCFH-DA assay was performed to evaluate ROS generation. Magnification: 200x. (d) Relative quantification of ROS in (c) was calculated. Data are represented as the mean ± SD of three independent experiments. ^∗^*P* < 0.05 versus (a) hNOKs or versus (b) control group. Ctrl: control; Mel: melatonin; NAC: N-acetyl-L-cysteine.

Cancer cells produce excessive ROS [7], and ROS has been implicated in oral cancer development [30]. To determine the levels of ROS in oral cancer cells, ROS production was measured with DCFH-DA kits. As shown in Figures 1(a) and 1(b), the generation of ROS was significantly increased in the five oral cancer cell lines (SCC25, SCC9, Tca8113, Cal27, and FaDu) compared with that in hNOKs. Cal27 and FaDu cells which had the highest ROS levels were selected for the following experiments. Melatonin exerts antioxidative effects in many cancer cells [27]. Consistently, melatonin markedly reduced ROS production in Cal27 and FaDu cells. And the free radical scavenger NAC was used as the positive control (Figures 1(c) and 1(d)). These results indicated that melatonin suppresses ROS generation in oral cancer cells.

### 3.2. Melatonin Reduces the Proliferation and Induces the Apoptosis of Oral Cancer Cells by Inactivating ROS-Dependent Akt Signaling

**Figure 2:**
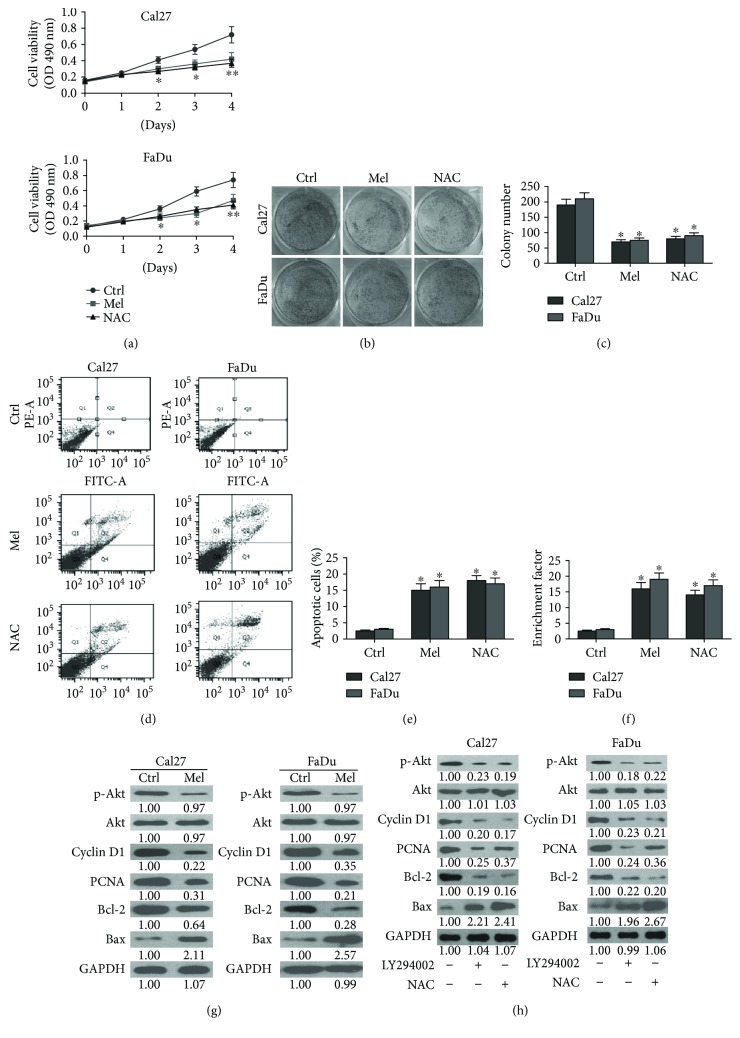
Melatonin inhibited the proliferation and enhanced the apoptosis of oral cancer cells via inactivation of ROS-dependent Akt pathway. (a–f) Cal27 and FaDu cells were exposed to melatonin (1 mM), NAC (5 mM), or the vehicle (control) for 24 h. (a) MTT assay was performed to measure the viability of Cal27 and FaDu cells. (b) Colony formation assay was conducted to determine the proliferation of Cal27 and FaDu cells. (c) The colony numbers were calculated. (d) Flow cytometry was carried out to analyze the apoptosis of Cal27 and FaDu cells. (e) The percentage of apoptotic cells was calculated. (f) Cell apoptosis was evaluated by enrichment factor assay. (g) Cal27 and FaDu cells were treated with melatonin (1 mM) or vehicle (control) for 24 h. Western blot was performed to detect the expressions of p-Akt, Akt, cyclin D1, PCNA, Bcl-2, and Bax. GAPDH was used as endogenous control. (h) Cal27 and FaDu cells were treated with LY294002 (20 *μ*M), NAC (5 mM), or the vehicle (control) for 24 h. Representative Western blot results of p-Akt, Akt, cyclin D1, PCNA, Bcl-2, and Bax. GAPDH was used as endogenous control. The ratios from the indicated proteins to GAPDH are indicated below the bands. Data are represented as the mean ± SD of three independent experiments. ^∗^*P* < 0.05 versus control group. Ctrl: control; Mel: melatonin; NAC: N-acetyl-L-cysteine. ^∗∗^*P* < 0.01 versus control group.

Excessive cell proliferation and apoptosis resistance are necessary for oral carcinogenesis [31]. Here, we found that melatonin substantially decreased the viability (Figure 2(a)) and proliferation (Figures 2(b) and 2(c)) of Cal27 and FaDu cells. As shown in Figures 2(d)–2(f), melatonin markedly increased the apoptosis of Cal27 and FaDu cells. Molecular analyses showed that melatonin reduced the expressions of prosurvival proteins including cyclin D1, PCNA, and Bcl-2 and enhanced the proapoptotic protein Bax level in Cal27 and FaDu cells (Figure 2(g)). Reportedly, Akt inactivation is responsible for proliferation inhibition and apoptosis elevation of oral cancer cells [9]. As shown in Figure 2(g), melatonin suppressed the activation of Akt in Cal27 and FaDu cells. Also, treatment with PI3K/Akt inhibitor LY294002 decreased the levels of cyclin D1, PCNA, and Bcl-2 but increased the expression of Bax in Cal27 and FaDu cells, paralleling with melatonin (Figure 2(h)). ROS contributes to proliferation of gastric cancer cells via upregulation of p-Akt [8]. Thus, we investigated whether ROS-dependent Akt activation was involved in the survival reduction of oral cancer cells by melatonin.  Figure 2(h) showed that ROS scavenger NAC treatment reduced the phosphorylation of Akt and the levels of cyclin D1, PCNA, and Bcl-2 and enhanced the expression of Bax. These results demonstrated that melatonin reduces the proliferation and apoptosis evasion of oral cancer cells by inhibiting ROS-activated Akt pathway.

### 3.3. Melatonin Suppresses the Mobility of Oral Cancer Cells via Inactivation of ROS-Reliant Akt Signaling

**Figure 3:**
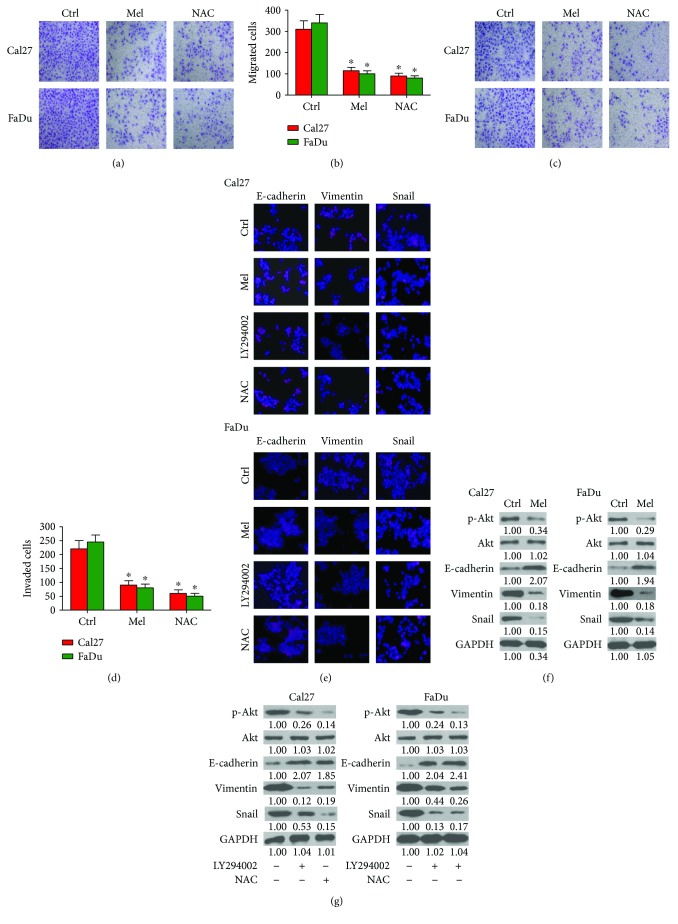
Melatonin abated the migration and invasion of oral cancer cell by inhibiting ROS-activated Akt signaling. (a–d) Cal27 and FaDu cells were exposed to melatonin (1 mM), NAC (5 mM), or the vehicle (control) for 24 h. Transwell assays were performed to assess the (a) migration and (c) invasion of Cal27 and FaDu cells. The number of (b) migrated cells and (d) invaded cells was calculated. (e) Cal27 and FaDu cells were exposed to melatonin (1 mM), LY294002 (20 *μ*M), NAC (5 mM), or the vehicle (control) for 24 h. Immunofluorescence staining was conducted to analyze the expression of E-cadherin, Vimentin, and Snail. Magnification: 100x. (f) Cal27 and FaDu cells were treated with melatonin (1 mM) or vehicle (control) for 24 h. Western blot was performed to detect the expressions of p-Akt, Akt, E-cadherin, Vimentin, and Snail. GAPDH was used as endogenous control. (g) Cal27 and FaDu cells were treated with LY294002 (20 *μ*M), NAC (5 mM), or the vehicle (control) for 24 h. Representative Western blot results of p-Akt, Akt, Snail, E-cadherin, and Vimentin. GAPDH was used as endogenous control. The ratios from the indicated proteins to GAPDH are indicated below the bands. Data are represented as the mean ± SD of three independent experiments. ^∗^*P* < 0.05 versus control group. Ctrl: control; Mel: melatonin; NAC: N-acetyl-L-cysteine.

Cell migration and invasion are important features of tumor metastasis [31]. Melatonin is reported to effectively repress the mobility of oral squamous carcinoma cells [32]. Transwell assay showed that melatonin significantly abolished the migration of Cal27 and FaDu cells (Figures 3(a) and 3(b)). Invasion of Cal27 and FaDu cells was also abated by melatonin treatment (Figures 3(c) and 3(d)). Molecularly, melatonin increased the expression of the epithelial cell marker E-cadherin and decreased the level of mesenchymal cell marker Vimentin and Snail (Figures 3(e) and 3(f)). Reportedly, Akt activation induces EMT and promotes the motility of oral cancer cells [33]. As shown in Figure 3(f), Akt was highly activated in Cal27 and FaDu cells, and melatonin reduced the phosphorylation of Akt and the level of Vimentin and Snail but increased the expression of E-cadherin. Similar to the effects of melatonin, treatment with PI3K/Akt inhibitor LY294002 led to substantial upregulation of E-cadherin and concomitant downregulation of p-Akt, Snail, and Vimentin (Figures 3(e) and 3(g)). Thus, melatonin represses the migration and invasion of oral cancer cells by suppressing activated Akt-triggered EMT. Next, we explored whether Akt activation-prompted EMT was ROS-dependent in oral cancer cells. Treatment with the ROS scavenger NAC reduced the phosphorylation of Akt and impaired the EMT of Cal27 and FaDu cells (Figures 3(e) and 3(g)). These findings indicated that inactivation of ROS-dependent Akt pathway is required for melatonin-decreased migration and invasion of oral cancer cells.

### 3.4. Melatonin Represses Proangiogenesis of Oral Cancer Cells through Inactivation of ROS-Dependent ERK and Akt Pathways

**Figure 4:**
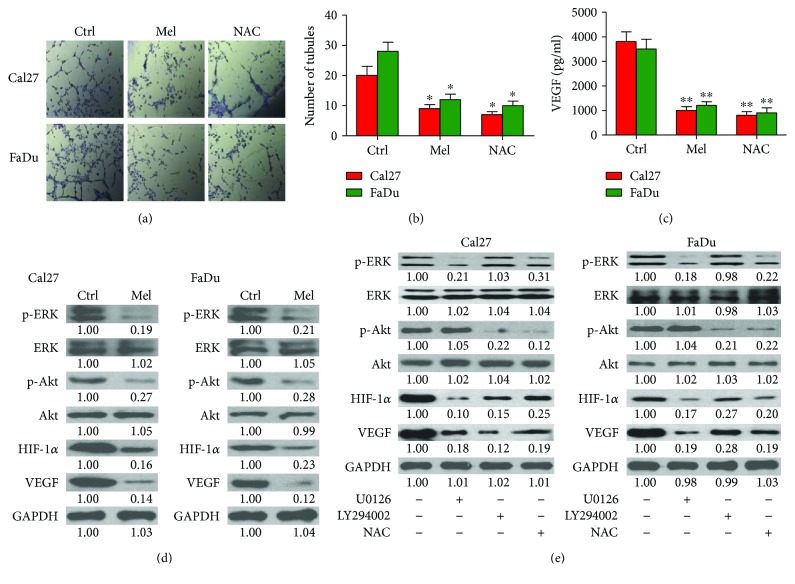
Melatonin suppressed vasculogenic mimicry of oral cancer cells by blocking ROS-dependent ERK/Akt signaling. (a and b) Cal27 and FaDu cells were treated with melatonin (1 mM), NAC (5 mM), or vehicle (control) for 24 h. CMs were harvested to incubate HUVECs for 36 h. (a) Representative images of vascular mimicry. (b) The number of tubules was calculated. (c) Cal27 and FaDu cells were treated with melatonin (1 mM), NAC (5 mM), or vehicle (control) for 24 h. CMs were collected for VEGF measurement by using ELISA assay. (d) Cal27 and FaDu cells were treated with melatonin (1 mM) or vehicle (control) for 24 h. Representative Western blot results of p-Ak, Akt, p-ERK, ERK, HIF-1*α*, and VEGF. GAPDH was used as endogenous control. (e) Cal27 and FaDu cells were incubated with U0126 (20 *μ*M), LY294002 (20 *μ*M), NAC (5 mM), or the vehicle (control) for 24 h. The expression of p-ERK, ERK, p-Akt, Akt, HIF-1*α*, and VEGF was measured by Western blot assays. GAPDH was used as endogenous control. The ratios from the indicated proteins to GAPDH are indicated below the bands. Data are represented as the mean ± SD of three independent experiments. ^∗^*P* < 0.05, ^∗∗^*P* < 0.01 versus control group. CMs: conditional medium; Ctrl: control; Mel: melatonin; NAC: N-acetyl-L-cysteine.

Melatonin reduces angiogenesis in colon cancer cells [34]. To examine whether melatonin has antiangiogenic effect on oral cancer cells, Cal27 and FaDu cells were treated with or without melatonin for 24 h, and then the CMs were collected to incubate HUVECs for tube formation assays. As shown in Figures 4(a) and 4(b), CMs from melatonin- or NAC-treated Cal27 and FaDu cells resulted in less tube-like structures than those in the control group. Moreover, melatonin or NAC treatment markedly abolished VEGF release from Cal27 and FaDu cells (Figure 4(c)). Therefore, melatonin abates oral cancer cell-induced angiogenesis. ERK and Akt activation is involved in angiogenesis during the malignant transformation of human bronchial epithelial cells [21]. The phosphorylation of ERK and Akt and the expressions of HIF-1*α* and VEGF were high in Cal27 and FaDu cells, whereas melatonin considerably decreased the levels of p-ERK, p-Akt, HIF-1*α*, and VEGF (Figure 4(d)). To investigate whether melatonin-suppressed vasculogenic mimicry was dependent on ERK and Akt signaling, the ERK inhibitor U026 and PI3K/Akt inhibitor LY294002 were employed. As shown in Figure 4(e), the expression of proangiogenic molecules HIF-1*α* and VEGF was remarkably downregulated in the presence of U026 or LY294002. Previously, the induction of HIF-1*α* was involved in ROS-ERK/Akt pathway [21]. ROS scavenger NAC led to downregulation of HIF-1*α* and VEGF in Cal27 and FaDu cells (Figure 4(e)). The data suggested that melatonin reduces proangiogenesis of oral cancer cells by inactivation of ROS-dependent ERK and Akt pathways.

### 3.5. Melatonin Retards Oral Cancer Tumorigenesis In Vivo

**Figure 5:**
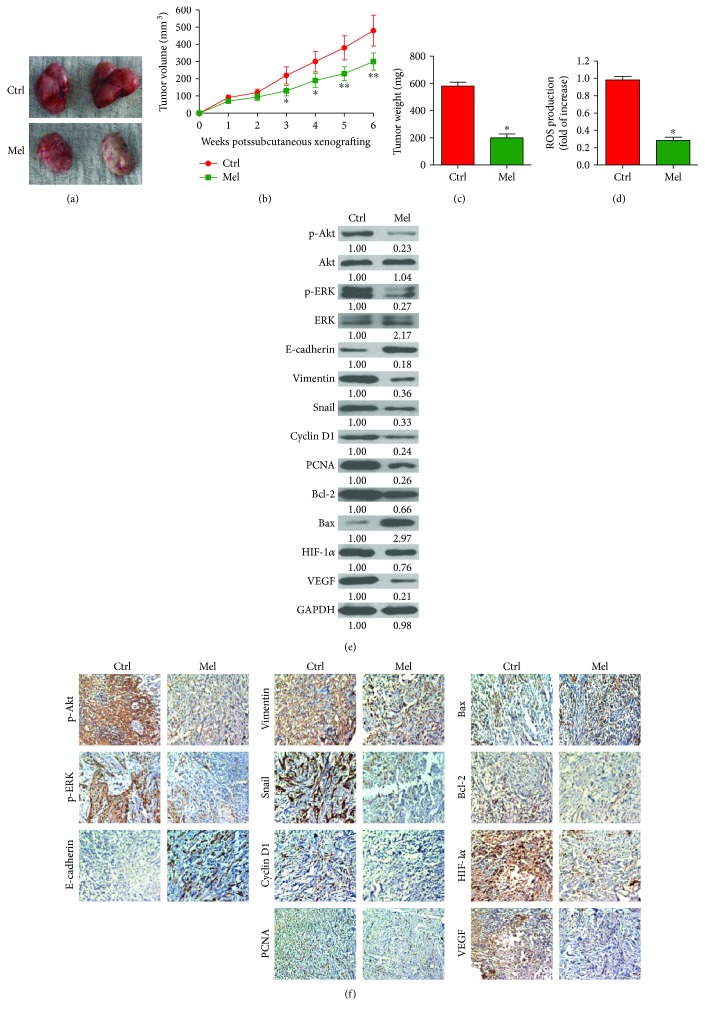
Melatonin hampered oral cancer tumorigenesis *in vivo*. SCID mice were subcutaneously injected with 2 × 10^6^ Cal27 cells. After 7 days, the animals received intraperitoneally melatonin or the vehicle (control) five days a week in a span of three weeks. All the mice were sacrificed under euthanasia six weeks after tumor cell implantation. (a) Representative images of tumors from the two groups. (b) Tumor volume was measured and calculated once a week for six weeks. (c) Tumor weight was measured. (d) ROS level was measured in the tumor tissues. (e) Western blot results of p-Akt, Akt, p-ERK, ERK, E-cadherin, Vimentin, cyclin D1, PCNA, Bcl-2, Bax, Snail, HIF-1*α*, and VEGF in the tumor tissues. GAPDH was used as endogenous control. The ratios from the indicated proteins to GAPDH are indicated below the bands. (f) IHC assays were performed to evaluate the levels of p-Akt, p-ERK, E-cadherin, Vimentin, cyclin D1, PCNA, Bcl-2, Bax, Snail, HIF-1*α*, and VEGF in the tumor tissues. Data are represented as the mean ± SD of three independent experiments. ^∗^*P* < 0.05, ^∗∗^*P* < 0.01 versus control group. Ctrl: control; Mel: melatonin; NAC: N-acetyl-L-cysteine.

To probe whether melatonin hampers oral cancer tumorigenesis *in vivo*, a xenograft mouse model was established by implanting Cal27 cells, and then the SCID mice were treated with melatonin or the vehicle. Melatonin-treated mice showed smaller tumors than those in the control mice (Figure 5(a)). The tumors grew slowly in the mice with melatonin administration (Figure 5(b)). Melatonin treatment also reduced tumor weight compared with the control group (Figure 5(c)). ROS production in the tumor tissues was markedly reduced in melatonin-exposed group compared to the vehicle group (Figure 5(d)). Molecular analysis of the tumor tissues showed that melatonin decreased the levels of p-Akt, p-ERK, cyclin D1, PCNA, Bcl-2, Vimentin, Snail, HIF-1*α*, and VEGF, but upregulated the expressions of Bax and E-cadherin (Figures 5(e) and 5(f)). These results indicated that melatonin hinders the tumorigenesis of oral cancer *in vivo*.

## 4. Discussion

In this study, melatonin represses oral cancer *in vitro* and *in vivo*. Key findings were as follows: first, melatonin suppressed ROS production in oral cancer cells. Second, melatonin reduced proliferation and apoptosis escape of oral cancer cells by inactivating ROS-dependent Akt signaling. Third, the inhibitory effect of melatonin on oral cancer cell migration and invasion is mediated by inactivation of ROS-reliant Akt signaling. Fourth, melatonin abolished tube formation of HUVECs induced by oral cancer cells by the blockage of ROS-activated Akt and ERK pathways. Lastly, melatonin hampered oral cancer tumorigenesis *in vivo*, which was consistent with *in vitro* findings. Overall, melatonin reduces ROS-driven proliferation, EMT, and vasculogenic mimicry in oral cancer, suggesting that melatonin may be a potential agent for oral cancer therapy.

ROS is highly involved in the cancer progression, which is a complicated process that includes uncontrolled proliferation and apoptosis resistance, migration and invasion of tumor cells, and angiogenesis around the tumor lesion [31, 35]. Previous studies demonstrated that ROS was excessively produced in oral cancer [36, 37]. Accordingly, we found that high amounts of ROS were generated in oral cancer cells and tumor xenografts. Experimental and clinical studies have illustrated the beneficial effects of melatonin in many human cancers, such as breast cancer, colorectal cancer, lung cancer, melanoma, and leukemia [38]. The anticarcinogenic effect of melatonin is positively associated with its antioxidative action [39]. In the present study, melatonin markedly reduced ROS production in oral cancer cells *in vitro* and *in vivo*, suggesting that melatonin elicits antioral cancer effects by inhibiting ROS generation.

Enhanced proliferation and resistance to apoptosis are vital features of human cancer [31]. The high expression of typical biomarkers of cell proliferation, such as cyclin D1 and PCNA, induces the propagation of unrepaired DNA damage, accumulation of genetic errors and a selective growth advantage for altered cells, thereby promoting oral cancer progression [40, 41]. Increased Bcl-2 and decreased Bax are frequently observed in oral cancer [42]. Increasing evidence confirmed that aberrant Akt activation promotes the proliferation and inhibits the apoptosis of oral cancer cells and regulates cell metabolic pathways required for tumor growth [42, 43]. Moreover, blocking PI3K/Akt signaling causes more oral cancer cells to undergo apoptosis [44]. In literature, melatonin reduces the proliferation and induces the apoptosis of oral cancer cells [45]. Consistent with these reports, we found that melatonin significantly reduced the proliferation and apoptosis resistance of oral cancer cells by downregulating the expressions of cyclin D1, PCNA, and Bcl-2 and upregulating Bax expression via inactivating Akt *in vitro* and *in vivo*. Increased ROS induces the phosphorylation of Akt, thereby leading to cancer cell proliferation and apoptosis evasion [8, 9]. In this study, treatment with ROS scavenger NAC reduced the levels of p-Akt, cyclin D1, PCNA, and Bcl-2 and increased the expression of Bax in oral cancer cells. Collectively, these results suggested that melatonin hinders the proliferation and triggers the apoptosis of oral cancer cells by inactivating ROS-dependent Akt signaling.

Local and distant metastases are the main causes of poor outcome of oral cancer [5]. EMT, a process whereby epithelial cells shift toward the mesenchymal phenotype and become highly mobile, is proposed to influence cancer cell migration, invasion, and metastatic dissemination [46]. Loss of epithelial markers, such as E-cadherin, and gain of the mesenchymal markers, such as Vimentin and Snail, are the hallmarks of EMT [47]. Akt is activated in human oral squamous carcinoma cells; constitutively activated Akt downregulates the level of E-cadherin and upregulates Vimentin and *β*-catenin expression [33]. Consistently, in this work, Akt activation was parallel with the increase of Snail and Vimentin and the decrease of E-cadherin in oral cancer cells. Treatment with Akt inhibitor LY294002 enhanced E-cadherin expression and reduced Snail and Vimentin levels, indicating that Akt activation-induced EMT promotes oral cancer cell migration and invasion. In the literature, ROS facilitates EMT via activation of Akt in colorectal cancer cells [16]. In this study, ROS scavenger NAC treatment led to the increase in E-cadherin expression and the decrease in p-Akt, Snail, and Vimentin levels in oral cancer cells. Melatonin inhibits the metastasis of a series of cancers, including oral cancer [32, 48]. In this report, melatonin abated the migration and invasion of oral cancer cells, downregulated the expression of p-Akt, Vimentin, and Snail, and upregulated E-cadherin level *in vitro* and *in vivo*. All the data suggested that melatonin may suppress oral cancer metastasis by reducing the ROS-dependent Akt activation.

Angiogenesis is a fundamental requirement for cancer metastasis. VEGF is the key growth factor that stimulates angiogenesis [49]. HIF-1*α* is a crucial transcriptional activator of VEGF expression by binding to its promoter [50]. HIF-1*α* increases tumor angiogenesis by inducing VEGF and other proangiogenic factors that contribute to a highly aggressive tumor phenotype [51]. ERK and PI3K/Akt pathways are essential for cancer angiogenesis [52]. ERK and Akt activation enhances HIF-1*α* and VEGF expression in human malignant lung epithelial cells and prostate cancer cells [21, 53]. In this study, we demonstrated that the phosphorylation of ERK and Akt and the expression of HIF-1*α* and VEGF were highly elevated in oral cancer cells *in vitro* and *in vivo*. Treatment with ERK inhibitor U0126 and Akt inhibitor LY294002 significantly reduced the levels of HIF-1*α* and VEGF, suggesting that the upregulation of HIF-1*α* and VEGF is partly dependent on ERK and Akt activation in oral cancer cells. Previously, ROS is involved in the activation of ERK and Akt and responsible for HIF-1*α* and VEGF expression [21, 53]. Melatonin suppresses angiogenesis by inhibiting ROS production to reduce HIF-1*α* stabilization in colon cancer cells [34]. Melatonin also inhibits HIF-1*α* expression in oral cancer cells [54]. In the present study, the levels of p-ERK, p-Akt, HIF-1*α*, and VEGF were remarkably decreased by ROS scavenger NAC or melatonin treatment. Melatonin markedly hampered tube formation of HUVEC and VEGF release by oral cancer cells. These results noted that melatonin suppresses angiogenesis relying on ROS-dependent ERK and Akt signaling in oral cancer cells, which involves HIF-1*α*.

The validity of melatonin as a prominent, naturally occurring oncostatic agent is examined in terms of its putative oncostatic mechanism of action, the correlation between melatonin levels and neoplastic activity, and the outcome of therapeutically administered melatonin in clinical trials. In literature, Lerner and Nordlund [55] reviewed the clinical pharmacology of melatonin and stated that “The person to ever receive 200 mg melatonin intravenously daily for 5 days has no evidence of delayed toxicity 18 years later.” This dose is the equivalent of, at a conservative estimate, the melatonin content of about a million human pineals daily [56]. Melatonin seems to be remarkably nontoxic and in experiments on rats, it was impossible to find an oral LD_50_, even with doses as high as 3200 mg/kg [57]. In humans, 250 mg melatonin taken orally every 6 hours for 25–30 days showed no toxic effects on the blood pressure, pulse rate, ECG, fundoscopy, full blood count, electrolytes, or liver enzymes, and the only side effect found was drowsiness [58]. Di Bella et al. [59] did report success with melatonin therapy in patients with lymphoma, leukemia, breast, stomach and lung cancer, and osteosarcoma. Lissoni and colleagues conducted a trial in which 20 mg intramuscular melatonin injections were given at 15 h daily to patients with metastatic solid tumors, and the malignancies were significantly improved [60]. According to the previous reports, we speculated that the experimental concentration of melatonin (40 mg/kg) in this study possibly has no toxic effects on physiological parameters and could become clinically achievable. However, further clinical studies are needed to verify the speculation.

## 5. Conclusion

In summary, melatonin abolishes ROS-promoted growth, metastasis, and angiogenesis in oral cancer. We found that melatonin suppresses ROS production in oral cancer cells. Melatonin inhibits the proliferation and induces the apoptosis of oral cancer cells. Melatonin reduces the migration and invasion of oral cancer cells by the repression of EMT. Moreover, melatonin decreases the angiogenesis of oral cancer. Molecular mechanistically, melatonin exerts antisurvival and antimobility effects by inactivating ROS-dependent Akt signaling and exhibits its antiangiogenesis function by blocking the ROS-activated ERK and Akt pathways in oral cancer. Together, these results indicate that reducing ROS by melatonin probably provides promising therapeutic protection against oral cancer.
